# Phenotypic Characters and Inheritance Tendency of Agronomic Traits in F1 Progeny of Pear

**DOI:** 10.3390/plants14101491

**Published:** 2025-05-16

**Authors:** Xiaojie Zhang, Mengyue Tang, Jiamei Li, Yue Chi, Kexin Wang, Jianying Peng, Yuxing Zhang

**Affiliations:** College of Horticulture, Hebei Agricultural University, Baoding 071000, China; zhangxiaojietyy@163.com (X.Z.);

**Keywords:** pear, hybrid progeny, fruit traits, genetic variation

## Abstract

Studying fruit genetic trends, heterosis, and growth traits in pear hybrid progeny provides the foundation for variety breeding. The aim of this research is to reveal the trait performance of the hybrid progeny of Chinese white pear and Western pear and provide a theoretical basis for other breeders to predict the trait performance of their hybrid progeny when selecting Eastern pear and Western pear as parents. Our research team constructed a ‘Yuluxiang’ × ‘Xianghongli’ interspecific hybrid population in 2015, and in 2023, we conducted a two-year investigation of 16 traits in 140 hybrid progeny, including 11 fruit traits and 5 growth traits, and analyzed and compared the genetic variation and heterosis of traits, as well as the correlation between various traits. The results showed that the hybrid progeny was widely segregated for single fruit weight (FW), soluble solid (SS) content, and titratable acid (TA) content and conformed to a normal distribution, with quantitative genetic traits under polygenic control. The highest two-year coefficients of variation for TA were 54.42% in 2023 and 39.17% in 2024. A genetic trend of decreasing FW was observed, which was greatly influenced by the male sex. The ratio of soft soluble flesh to crispy flesh was 1:1, and the gene controlling this trait may be a quality trait controlled by a single gene. The traits that showed transgressive heterosis for two years included fruit longitudinal diameter (FLoD), fruit shape index (FSI), and TA, and those that showed negative heterosis included FW, SS, leaf longitudinal diameter (LLoD), and leaf lateral diameter (LLaD). Correlation analysis indicated that the progeny of crosses in this combination, which had red fruit skin, may also present red early flowering color (EFC) and young leaf color (YLC), reddish brown annual branch color (ABC), and lower FSI, fruit stalk length (FSL), LLaD, and TA. Thus, at the seedling stage, individuals with red-colored fruit may be screened by observing the color of young leaves and young stems and the lateral diameter of the leaves. Principal component analysis showed that among the 16 traits included in six principal components, peel color (PC), FLoD, 2024SS, fruit tape (FT), and FSI were the main factors causing differences in fruit phenotypes. This study systematically elucidated the genetic trends of agronomic traits in pears and will provide a theoretical basis for the selection of parents and early selection of hybrid progeny in pear hybrid breeding.

## 1. Introduction

Pears are one of the world’s major fruits, and they are loved by consumers because of their unique taste and benefits to human health [1]. The world’s pear cultivars can be divided into two categories: Eastern and Western [2]. Oriental (Eastern) pears, including white, sand, Akiko, and Xinjiang pears, are primarily cultivated in China, Japan, and South Korea. Western pear cultivation is more extensive, with Europe, North America, South America, Oceania, and South Africa representing the five production areas. In 1870, American missionaries first spread pear to Yantai in Shandong, and then Western pear cultivation spread to the mainland. The pear cultivation area in Hebei Province ranks among the highest in China. This region is an advantageous pear production area that has broad development prospects.

Variety breeding represents an ongoing domestic and international research topic and a hot topic in global fruit tree research [3]. Pear crossbreeding is the main breeding method because it can quickly integrate the genomes of different seed sources to obtain new varieties with better performance; however, the breeding cycle is long [4]. In recent years, Chinese breeders have produced some high-quality varieties by crossbreeding, such as ‘Huatong’ [5], ‘Xingli Yanhong’ [6], and ‘Mingjing’ [7]. However, the main plant varieties present a single structure and thus cannot meet the various needs of consumers. Therefore, there is an urgent need to cultivate better pear varieties to meet consumer demands.

Knowledge of genetic variations in important traits is the basis of crossbreeding, which can provide theoretical guidance for the selection of parents and early selection of hybrid progeny in pear hybrid breeding. The pear genome is highly heterozygous because of the fruit’s self-incompatibility trait, and hybrid progeny tends to exhibit a wide segregation of traits [8]. Most of the pear traits are quantitative traits, which are generally considered to be controlled by a large number of polygenes. However, this assumption is inconsistent with many studies. For example, QTL (quantitative trait locus) localization analysis results have shown that major genes and polygene mixed inheritance control the inheritance of quantitative traits when large differences are observed in effect size [9]. So, it is very difficult to study the genetic laws of pear traits. The research on the genetic characteristics of pear traits in European countries has mainly focused on Western pears. Dondini’s three-year study of seven pear hybrid combinations revealed the genetic laws of red traits and the fact that red traits are dominant traits controlled by a single gene [10]. Sewon used an interspecific hybrid population (*Pyrus pyrifolia* × *P. communis* × *P. pyrifolia*) to study resistance to pear scab, and the segregation ratio of resistance in the hybrid offspring was 1:1, indicating that this trait is a dominant inheritance controlled by a single gene [11]. The research on the genetic characteristics of pear traits in Asian countries has mainly focused on Eastern pears. Hwang’s study of fruit texture traits using 15 Eastern pear hybrid combinations revealed that fruit hardness is inherited towards the middle value of the parents [12]. At present, there is relatively little research on the hybrid combination of Eastern pears and Western pears. Liu used reciprocal crossing populations ‘Dangshansuli’ and ‘Housui’ and revealed that the content of organic acids in the progeny was more influenced by the female parents [13]. Trait inheritance trends vary in other species. For example, in terms of titratable acid inheritance, plums are influenced more by their male parents [14], while blueberries are influenced more by their female parents [15]. This shows that the inheritance of fruit tree traits is complex. Previous studies have mostly analyzed the inheritance of a single trait or a few traits, without a comprehensive study of multiple traits, so a more comprehensive study of important agronomic traits of pears is still necessary.

In order to cultivate new high-quality storage-resistant red varieties, in 2015, our team used the Chinese white pear variety ‘Yuluxiang’ as the female parent [16] and the Western pear variety ‘Xianghongli’ as the male parent [17] and successfully obtained 900 hybrid progeny. In 2023 and 2024, we explored the traits of those hybrid progeny. Genetic, heterosis, and correlation analyses were conducted on the main traits of the F1 generation to explore whether there was a linkage inheritance phenomenon for each trait. These results will provide a reference for pear parent selection and lay a theoretical foundation for future in-depth research on QTL mapping of major pear traits [18] and molecular marker-assisted breeding [19].

## 2. Materials and Methods

### 2.1. Plant Materials

The plant materials were from the F1 interspecific hybrid population of the Western pear ‘Xianghongli’ and Chinese white pear ‘Yuluxiang’, which were planted at the pear breeding base in Xingtai City, Hebei Province, China, in 2017. This region has a temperate monsoon climate, with an average annual temperature ranging from 12 °C to 14 °C. The soil type is sandy loam, and the irrigation method is drip irrigation. The precipitation in 2023 was 677.7 mm, and in 2024, it was 562.1 mm. The flowering periods in these two years were consistent. Under the conventional management mode with a spacing of 1 m × 4 m between plants and rows, approximately 148 F1 populations were surveyed in 2023 and 2024.

### 2.2. Data Collection and Analysis Methods

This study investigated and analyzed 11 fruit traits and 5 growth traits of hybrid progeny over two years. The fruit traits included fruit weight (FW), fruit longitudinal diameter (FLoD), fruit lateral diameter (FLaD), fruit shape index (FSI), fruit stalk length (FSL), total soluble solids (TSS), titratable acid (TA), soluble sugar (SS), fruit tape (FT), peel color (PC), and fruit heart size (FHS). The growth traits included leaf longitudinal diameter (LLoD), leaf lateral diameter (LLaD), young leaf color (YLC), early flowering color (EFC), and annual branch color (ABC).

According to the degree of peel color, the fruit was divided into five categories: 1, peel without color; 2, 1/4 peel with red; 3, 1/2 peel with red; 4, 3/4 peel with red; and 5, peel with full red. The color of early flowering was divided into 1 (white) and 2 (red). The color of the young leaves was divided into 1 (green) and 2 (red). The color of the annual branch was divided into 1 (yellowish brown) and 2 (reddish brown). Fruit type (FT) was divided into two categories: 1, soft soluble flesh that needs to be ripened, and 2, crispy flesh that does not need to be ripened. The classification method for traits was referenced from the book “Descriptors and Date Standard for Pear (*Pyrus* spp.)” [20].

At least 15 pears at different orientations were collected from each tree and were brought back to the laboratory for measurement. FW was measured using a balance, FLod, FLad, FSL, FHS, LLoD, and LLaD were measured using a vernier caliper, and YLC, EFC, and ABC were observed and statistically analyzed in the field. TSS was measured using PAL-1 from ATAGO company (Tokyo, Japan), TA was measured by sodium hydroxide titration [21], and SS was measured by anthrone colorimetry [22].

### 2.3. Statistical Analysis

Data from the two years were summarized and statistically analyzed. The minimum, maximum, mean (F), standard deviation (S), and coefficient of variation (CV/%) were calculated for each trait, with the CV calculated as CV/% = S/F. The Kolmogorov–Smirnov test was used to test the normal distribution of each trait (*p* < 0.05). The broad-sense heritability (*H*_b_^2^) for each trait was estimated according to the formula *H*_b_^2^ = [$\delta_{p}^{2}$ − ($\delta_{f}^{2}$ + $\delta_{m}^{2}$)/2]/$\delta_{p}^{2}$, where $\delta_{p}^{2}$ denotes the phenotypic variance of the F1 generation and $\delta_{f}^{2}$ and $\delta_{m}^{2}$ represent the phenotypic variance of the parents. Genetic transmission ability (*Ta/%*) was calculated as *Ta* = *F*/*MP*, where *F* is the mean value of the F1 generation and MP is the mid-parent value.

All data and tables were statistically analyzed using Excel, and Spearman’s correlation coefficient matrix between traits was calculated using SPSS 22.0. A one-sample mean *t*-test was used to determine whether differences were statistically significant (*p* < 0.01). Principal component analysis and normal distribution detection were performed using Prism software, and curve fitting was performed using nonlinear regression and a Gaussian model. Heterosis (Hm/%) of the traits was also analyzed based on the phenotype data collected above and calculated as Hm = (F − P)/MP. The OHP (over higher parent ratio) and BLP (below lower parent ratio) values were also summarized. OHP = X1/N, BLP = X2/N, where X1 is the number of plants with trait values over the higher parent, X2 is the number of plants with trait values below the lower parent, and N is the number of plants in the F1 generation of the test.

## 3. Results

### 3.1. Trait Differences Between Parents

**Figure 1:**
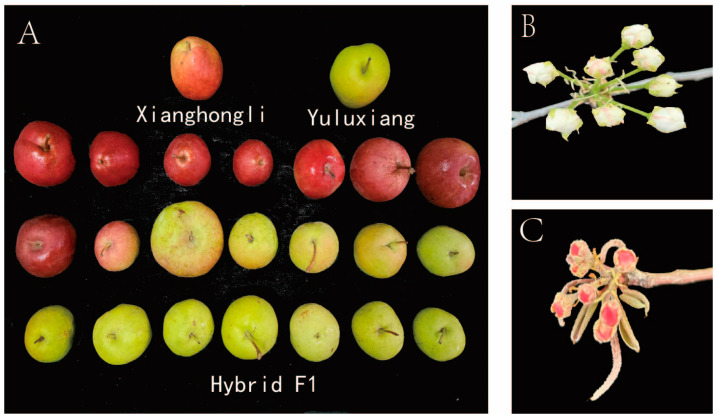
Yuluxiang’ × ‘Xianghongli’ hybrid F1 phenotype. Note: (**A**): fruit of the hybrid progeny; (**B**): early flowering of ‘Yuluxiang’; (**C**): early flowering of ‘Xianghongli’.

Figure 1 and Table 1 reveal that the traits between parents showed large differences. In 2023 and 2024, FW, FLod, FLad, FSL, TA, SS, LLod, and LLad showed significant differences. In 2023, TSS was not significantly different between parents, while in 2024, the TSS content of the female parent ‘Yuluxiang’ was significantly higher than that of the male parent ‘Xianghongli’. In fruit size and leaf size, ‘Yuluxiang’ was significantly higher than ‘Xianghongli’. Parental FHS performance was non-significant in both years. FSL was measured only in 2023, and it was significantly higher in ‘Yuluxiang’ than in ‘Xianghongli’. Among PC traits, the male parent ‘Xianghongli’ showed all red coloring while the female parent ‘Yuluxiang’ was only partially reddish (Figure 1A). Their progeny showed different degrees of pericarp coloration, with some showing all pericarp red coloring, partial pericarp red coloring, and all pericarp green coloring. The young leaves of ‘Yuluxiang’ and ‘Xianghongli’ were green and red, respectively; the colors of the annual branches of ‘Yuluxiang’ and ‘Xianghongli’ were yellowish brown and reddish brown, respectively. The early flowering colors of ‘Yuluxiang’ and ‘Xianghongli’ were white (Figure 1B) and red (Figure 1C), respectively. The above findings reveal that the parents are genetically distant and show large differences between several traits. These traits should be further genetically analyzed.

### 3.2. Inheritance Analysis of Traits

**Figure 2:**
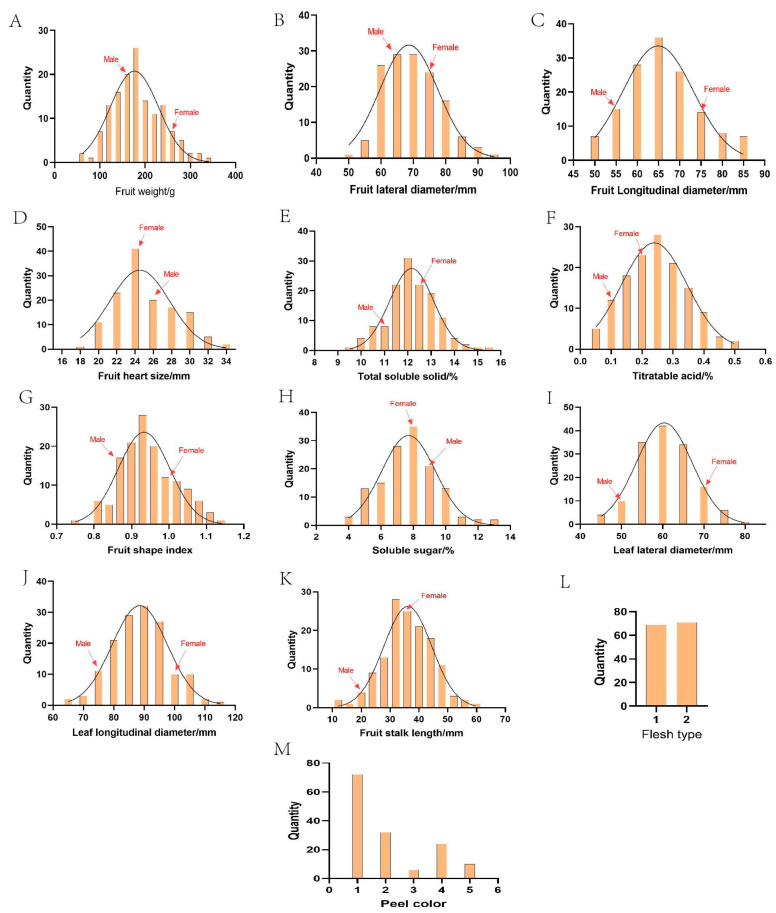
The segregation results of traits in the F1 population of ‘Yuluxiang’ and ‘Xianghongli’ in 2023.

**Figure 3:**
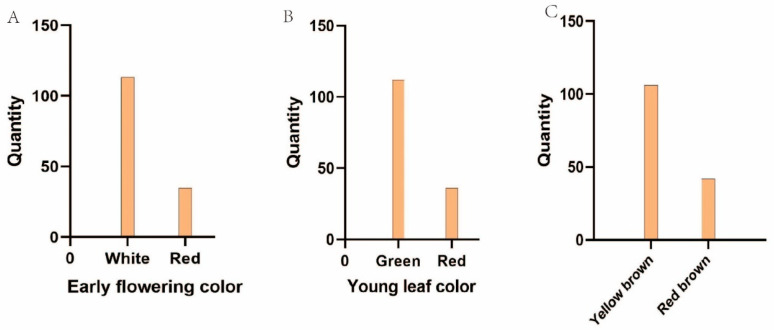
The segregation results of early flower color (**A**), young leaf color (**B**), and annual branch color (**C**) in the F1 population of ‘Yuluxiang’ and ‘Xianghongli’.

**Figure 4:**
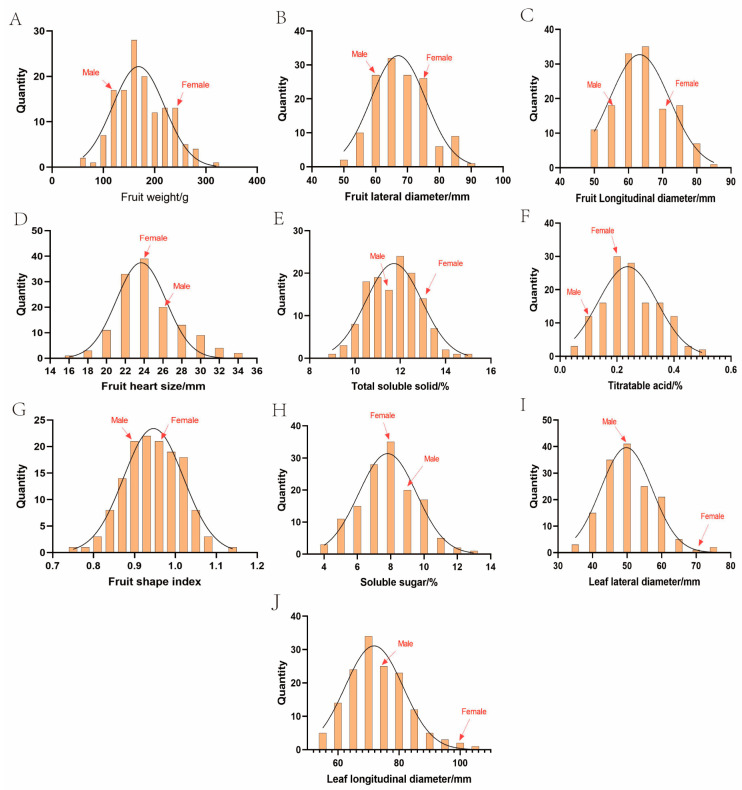
The segregation results of traits in the F1 population of ‘Yuluxiang’ and ‘Xianghongli’ in 2024.

As shown in Figure 2, Figure 3 and Figure 4, we tested for a normal distribution and graded the 16 traits. Eleven traits in 2023 (Figure 2A–K) and ten traits in 2024 (Figure 4A–J) showed widespread segregation, with varying degrees of a normal distribution (Table 2). The intervals where the male and female parents were located are marked on the map. The grading results of the five traits (Figure 2L,M and Figure 3) revealed that the FT of the F1 progeny was divided into two types, soft soluble pulp and crispy pulp, at a ratio of nearly 1:1. For the PC trait, the F1 progeny had the highest number of uncolored fruits, accounting for 50% of all F1 fruits. Half of the fruit peels had the smallest proportion of red, while 6.9% of the fruit peels were completely red. When all the colored fruit peels were classified into one category, the ratio was close to 1:1 compared to the non-colored fruit peels (Figure 2M). The F1 progeny EFC was mostly white, the YLC was mostly green, and the ABC was mostly yellow-brown (Figure 3).

According to the segregation statistics of the 11 traits in the F1 progeny (Table 2), 11 fruit and leaf traits showed different degrees of variation. The smallest CV in 2023 was 7.96% for FSI, while the largest was 54.42% for TA. The smallest CV in 2024 was 7.2% for FSI, while the largest was 39.17% for TA. The largest difference in the CV between the two years was for TA at 15.25%, and the smallest differences were for FLoD, FHS, and FLaD at 0.05%, 0.07%, and 0.25%, respectively. FW, TA, and SS varied by more than 20% in both years, indicating a wide separation of the traits. The CVs of TSS for the two years were relatively low at 8.69% and 9.51%.

Specifically, the F1 progeny FW in 2023 ranged from 71.93 g to 364.33 g, with a mean value of 185.10 g, and the FW in 2024 ranged from 57.80 g to 317.53 g, with a mean value of 174.38 g (Table 2). The FW in 2024 was overall smaller than that in 2023, which may have been related to environmental factors. In 2023 and 2024, the TSS content ranged from 9.70% to 15.50% and 8.94% to 14.80%, respectively. The mean value in 2024 was 11.72%, which was smaller than that in 2023 (12.17%). The TA content ranged from 0.03% to 0.55% and 0.05% to 0.49% in 2023 and 2024, respectively, with a mean value of 0.25%. The SS content ranged from 3.65% to 12.95% in 2023, with a mean value of 7.75% in 2023, and from 4.03 to 12.76% in 2024, with a mean value of 7.91%. The variations between the two years were relatively small, suggesting that the SS content was not easily influenced by the environment. The leaf size of the hybrid progeny varied widely, with the LLoD ranging from 62.84 to 114.31 mm in 2023 and from 53.93 to 103.76 mm in 2024 and the LLaD ranging from 45.29 to 79.29 mm in 2023 and 34.57 to 74.08 mm in 2024. In conclusion, in the F1 progeny, most quantitative traits were under polygenic control, which can be analyzed as the next step of the heterosis analysis.

### 3.3. Heterosis Analysis

As shown in Table 3, we analyzed 11 fruit and leaf traits for heterosis. The broad-sense heritability (*H*_b_^2^/%) values varied between traits and years for the same trait and ranged from 27.17% for FLoD to 85.42% for FSL in 2023 and from 32.31% for FLoD to 62.81% for LLoD in 2024. The *H*_b_^2^ value of FSL was the highest among the fruit traits, indicating that FSL is not easily affected by environmental factors. The *H*_b_^2^ values of FSI over the two years were 34.25% (2023) and 33.21% (2024), which were relatively low, indicating that fruit shape is more susceptible to environmental factors. The *H*_b_^2^ values of other fruit traits for the two years were as follows: FW, 60.47%, 55.23%; TSS, 48.83%, 52.91%; TA, 66.67%, 51.84%; SS, 57.38%, 49.25%; and FHS, 56.27%, 43.82%. The *H*_b_^2^ values of the leaf traits for the two years were as follows: LLoD, 69.22%, 62.81%; LLad, 61.27%, 55.47%. In 2023, the genetic transmission ability (Ta/%) of fruit traits ranged from 78.69% for FSL to 116.01% for SS, whereas in 2024, it ranged from 90.87% for SS to 159.24% for TA. In addition, the Ta/% for leaf traits for the two years had values of 101.07% and 65.05% for LLoD and 100.45 and 83.17 for LLaD (Table 3).

Heterosis rates varied considerably among the traits in the hybrid progeny (Table 3) and ranged from −13.80% for SS to 27.08% for FSL in 2023 and from −31.90% for FLaD to 59.24% for TA in 2024. The traits that had positive heterosis rates in both 2023 and 2024 were FLoD, FSI, and TA. For the two years, the TA heterosis rates were 23.65% and 59.24%, transgressive heterosis rates were 36.76% and 60.87%, and negative heterosis rates were 20.59% and 10.14%. This indicated that TA exhibited transgressive inheritance in this hybrid combination. Traits that did not show heterosis in 2023 and 2024 were FW, SS, LLoD, and LLaD. For the two years, the heterosis rates were −12.36% and −8.16%, transgressive heterosis rates were 9.29% and 11.35%, and negative heterosis rates were 54.29% and 22.70% for FW. This indicated a genetic trend towards smaller fruit sizes (Table 3). Interestingly, some progeny had FW values exceeding 300 g, indicating that new high-quality hybrid plants of the large-fruiting type could still be selected (Figure 2A and Figure 4A). The rates of heterosis for SS were −13.80% and −9.13%, and the rates of transgressive heterosis were 18.52% and 23.36%, which were less than the rates of negative heterosis (70.37% and 58.39%, respectively), indicating traits showing negative heterosis effects (Table 3). The mean values of FHS of the progeny in 2023 and 2024 were 25.09 mm and 24.57 mm, the MP values were 25.43 mm and 24.55 mm, and the rates of heterosis were −1.35% and 0.08%, respectively, thus indicating mid-parent inheritance (Table 3).

### 3.4. Trait Correlation Analysis

**Figure 5:**
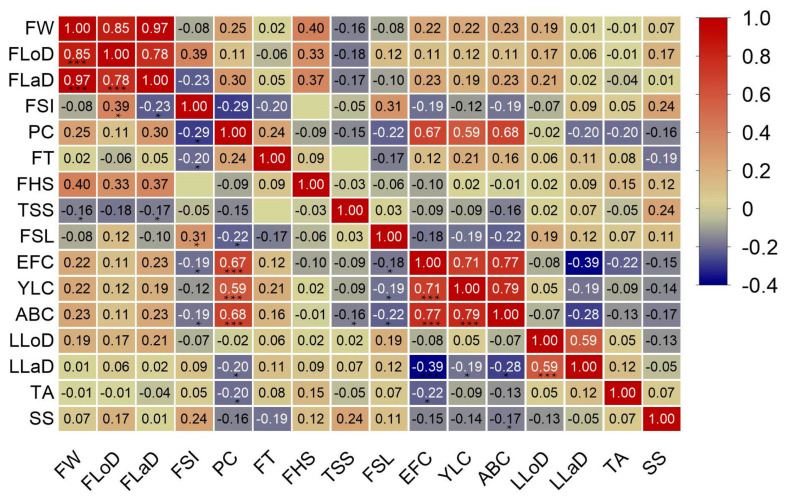
Spearman’s correlation matrix between 16 traits in the hybrid progeny. Note: * presents a significant correlation at 0.05; *** presents a highly significant correlation at 0.01.

The average of the two years of data was taken, and a correlation analysis was performed for 16 traits in the progeny (Figure 5). The results showed that FW was significantly and positively correlated with FLoD (0.85) and FLaD (0.97), which verified the accuracy of the correlation analysis (Figure 5). PC was significantly and positively correlated with EFC, YLC, and ABC, with correlation coefficients of 0.67, 0.59, and 0.68, respectively. PC was significantly negatively correlated with FSI, FSL, LLaD, and TA, with correlation coefficients of −0.29, −0.22, −0.20, and −0.20, respectively. Therefore, the hybrid progeny of this combination, which showed a red peel color, may also have a red EFC and YLC, a red-brown ABC, and smaller FSI, FSL, LLaD, and TA. We also found that LLaD was significantly negatively correlated with EFC, ABC, and YLC, with correlation coefficients of −0.39, −0.28, and 0.19, respectively, but was not significantly correlated with LLoD. FW was significantly negatively correlated with TSS content, suggesting that larger fruits had lower TSS contents.

### 3.5. Principal Component Analysis of Traits

**Figure 6:**
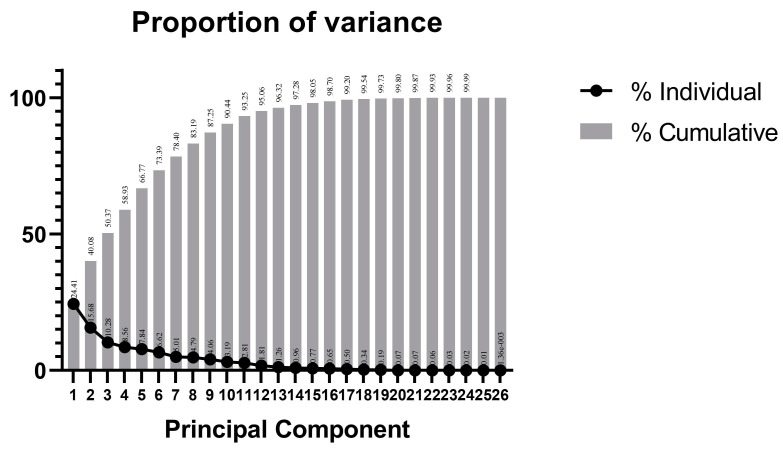
Variance proportion chart of each PC.

Principal component analysis of the 16 progeny traits was divided into six principal components, with a total contribution of 73.39% (Table 4, Figure 6). Principal component 1 accounted for the largest contribution (24.41%), followed by principal components 2, 3, 4, 5, and 6 (15.68%, 10.28%, 8.56%, 7.84%, and 6.62%, respectively). As shown in Table 5, the largest eigenvalues for principal components 1, 2, 3, 4, 5, and 6 were as follows: PC at 0.242; 2023FLoD at 0.525; 2023LLaD at 0.585 and 2024SS and 2024FLoD for fruit traits at 0.568; FT at 0.598; 2023LLoD at 0.592 and 2023FSI at 0.421 for fruit traits; and 2024FSI at 0.309, respectively (Table 5). The results showed that among the 16 traits included in the six principal components, PC, FLoD, 2024SS, FT, and FSI were the main factors causing the differences in fruit phenotypes of hybrid progeny as well as the main morphological indices for evaluating parental selection in pear breeding.

## 4. Discussion

Hybrid advantages are often utilized in fruit tree breeding to improve certain traits and breed new high-quality varieties [23]. The self-incompatibility characteristics of pears, which result in a highly heterozygous genome, also provide a good genetic basis for breeding pear hybrids [24]. In the past, breeders tended to select parents through empirical methods, which resulted in an inability to efficiently select new varieties that met the desired goals. With more and more hybrid combinations now being reported, breeders can more correctly select parents and predict progeny trait performance. The greater the differences in traits between parents, the more pronounced their hybrid advantages. In this study, the female ‘Yuluxiang’ and the male ‘Xianghongli’ are distantly related and show large differences between traits. Moreover, their progeny is widely segregated, with most of the traits showing normal distribution and some of the traits showing over higher or below lower parent progeny. Therefore, better varieties could be selected from the progeny. Single FW is a key indicator of pear fruit quality and yield and one of the traits most susceptible to environmental factors [25]. Single FW is a quantitative trait controlled by multiple genes [26]. In both years, the progeny single FW was genetically biased towards small fruits, and the rate of negative heterosis was greater than that of transgressive heterosis, which is consistent with the results of studies on cherry [27], *Castanea mollissima* [28], and loquat [29]. In 2023, the largest single FW of the hybrid progeny was 364.33 g, the smallest was 71.93 g, and the mean was 185.10 g. In 2024, the largest single FW was 317.53 g, the smallest was 57.80 g, and the mean was 174.38 g. The overall single FW in 2024 was lower than that in 2023, due to low precipitation and drought in 2024 [30]. The absence of fruit types larger than 400 g in the progeny of this hybrid combination may be due to the small fruit size of the male parent and the weak additive effect among genes [31].

Soluble solids are a key factor for determining the flavor of pear fruits; therefore, in the pear breeding process, breeders tend to use high TSS content as a key index for superior plant selection [32]. Luo’s study on soluble solids in jujube showed that the inheritance of the soluble solid content in jujube is a quantitative trait controlled by multiple genes, which is consistent with our results; however, the TSS content of jujube hybrid progeny tended to decrease [33]. In this study, the TSS content exhibited a normal distribution in 2023 and 2024. The highest TSS content in the progeny in 2023 was 15.50%, with a transgressive heterosis rate of 38.81%. The highest TSS content in the progeny in 2024 was 14.80%, with a transgressive heterosis rate of 12.32%. Overall, the two years showed a trend towards moderate inheritance, although some high-quality hybrid plants with high TSS content could still be selected. The *H*_b_^2^ value of TSS remained at approximately 50% for two years, indicating that this trait is susceptible to environmental influences, which is consistent with studies on peaches [34] and apricots [35]. In the actual breeding process, after superior plants are grafted, the TSS content can often be appropriately increased by optimizing cultivation conditions [36]. SS tends to be inherited from females and shows an overall decreasing trend, which is consistent with studies on apples [37]. In the F1 progeny, the TA content showed continuous variations with a normal distribution and the highest CV, indicating transgressive inheritance. This may be caused by additive effects between genes. Similar results have been observed in apples [38] and apricots [39]. The FSL transgressive heterosis rate was much greater than the negative heterosis rate in the progeny, which showed transgressive inheritance and relatively high *H*_b_^2^ values. These findings suggest that the FSL trait is mainly determined by genotypic effects and is less influenced by the environment, as observed in other hybrid combinations in pear [40].

Red fruits tend to be more popular among consumers [41]. Fruit skin color is a quantitative trait controlled by multiple genes, and several genes have been reported to regulate the mechanism of synthesis of pericarp anthocyanin glycerol, such as *PpbHLH64* [42], *PpERF9* [43], and *PpPIF8* [44]. In this study, the cross progeny were widely segregated in terms of peel coloration (Figure 1), with the highest number of fruits without coloration (accounting for 50% of all individuals), the smallest percentage of fruits with one-half peel with red color, and a low percentage of full red peel (6.9%). Dondini analyzed the red fruit traits of seven European pear hybrid combinations, and the results showed that when the parents had red fruit color, the ratio of red fruits to non-red fruits in their F1 was 3:1. When the parents had red and yellow fruit colors, the ratio of red fruits to non-red fruits in their F1 was 1:1 [10]. Xue used ‘Hongzaosu’ as a hybrid breeding parent; the ratio of red to green in their offspring was 1:1 [45]. This is consistent with the results of this study, indicating that the red fruit trait is a dominant trait. In addition, in terms of the inheritance of fruit type, the cross progeny had a 1:1 ratio of soft soluble and crisp flesh; moreover, this quality trait may be controlled by a single gene. Asian consumers prefer to consume crisp-fleshed pear fruits [46]. The trait correlation analysis in our study showed that the correlation coefficient between PC and FT was 0.24; moreover, an all-red peel color and crisp-fleshed flesh type were not observed in the hybrid progeny. These results showed that there was no linkage of inheritance between peel color and flesh type; thus, further backcrossing with the female parent is needed to breed varieties with full red peel color and crispy flesh type.

Certain correlations exist between various fruit tree traits. Liu analyzed the correlations of various cherry traits and showed that late-maturing Chinese cherries usually have longer developmental periods, larger fruits, and darker colors [47]. Alizadeh analyzed fruit characteristics of 19 pear varieties from northern Iran and showed that fruit soluble solids and soluble sugars were significantly positively correlated [48]. In our study, FW was significantly negatively correlated with TSS content, probably due to the association of fruit enlargement with cellular water content, which results in relatively low TSS content [49]. Some pear fruits show red varieties whose young leaves and stems are also red [50], which indicates the potential of screening varieties with red fruit in advance based on some early traits. Correlation analysis indicated that progeny of crosses in this combination with red fruit skin may present red EFC and YLC, reddish brown ABC, and lower FSI, FSL, LLaD, and TA. In other words, at the seedling stage, it is possible to screen for individuals whose fruits may exhibit red color by observing the color of young leaves and young stems and the lateral diameter of the leaves. In the future, a database may be constructed utilizing genome resequencing to predict the phenotypes of hybrid progeny more accurately; however, such work will require large populations and large amounts of phenotypic data [51]. With the development of genomics, the use of gene chips to predict apple color [52], ripening stage [53], and salinity tolerance [54] has been achieved.

In addition, we screened some superior plants with different fruit colors, flavors, and sizes using this hybrid combination, which is important for breeding new pear varieties [55]. Scientific selection of parents is important in breeding. Understanding the phenotypes of progeny from different hybrid populations by reviewing available data can help breeders select better parents and predict progeny phenotypes. In the selection of new high-quality hybrid plants, the phenotype of plants often does not reflect all traits that meet the expected goal; thus, hybrid plants can be used as the male parent and crossed with the female parent. Such work is expected to improve certain traits in superior plants and promote the innovative use of pear germplasm resources to provide a basis for directed breeding.

## 5. Conclusions

This study conducted a two-year genetic variation analysis and heterosis analysis on 11 fruit traits and 5 growth traits of the F1 hybrid population of ‘Yuluxiang’ and ‘Xianghongli’. The findings have revealed widespread segregation for traits such as single fruit weight, total soluble solids, and titratable acid, with richer genetic variation in titratable acid content. The progeny population conforms to a normal distribution and is a polygenetically controlled inheritance of a quantitative trait. Fruit flesh types (soft soluble flesh and crispy flesh) were probably inherited as qualitative traits controlled by a single gene. Titratable acid and fruit shape index showed transgressive inheritance, and single fruit weight and soluble sugar showed negative inheritance. The color of young leaves and young stems and the lateral diameter of leaves can be used as indicators for the early screening of red fruits. The generalizability of the findings of this study needs to be verified with more hybrid combinations. In the future, our work will focus on genetic linkage mapping and QTL localization using hybrid progeny with diverse progeny performance.

## Figures and Tables

**Table 1:** Phenotypic differences between the parents ‘Yuluxiang’ and ‘Xianghongli’.

Year		FW/g	FLoD/mm	FLaD/mm	FSI	FSL/mm	TSS/%	TA/%	SS/%	FT	PC	FHS/mm	LLoD/mm	LLaD/mm	YLC	EFC	ABC
2023	Female	256.96 a	71.63 a	75.07 a	0.95 a	37.95 a	12.53 a	0.20 a	8.68 a	2	2	24.76 a	104.32 a	72.16 a	1	1	1
	Male	165.47 b	56.33 b	63.75 b	0.88 b	18.66 b	11.20 a	0.11 b	9.29 b	1	5	26.11 a	75.20 b	50.15 b	2	2	2
2024	Female	243.52 a	70.21 a	72.35 a	0.97 a		13.02 a	0.20 a	8.24 a	2	2	23.39 a	101.58 a	70.31 a	1	1	1
	Male	136.23 b	54.36 b	60.68 b	0.90 b		11.47 b	0.11 b	9.17 b	1	5	25.71 a	74.31 b	51.64 b	2	2	2

Note: a, b indicate an extremely significant difference at a 0.01 probability level. The fruit traits included fruit weight (FW), fruit longitudinal diameter (FLoD), fruit lateral diameter (FLaD), fruit shape index (FSI), fruit stalk length (FSL), total soluble solids (TSS), titratable acid (TA), soluble sugar (SS), fruit tape (FT), peel color (PC), and fruit heart size (FHS). The growth traits included leaf longitudinal diameter (LLoD), leaf lateral diameter (LLaD), young leaf color (YLC), early flowering color (EFC), and annual branch color (ABC). The same below.

**Table 2:** Phenotypic distribution of certain traits in the F1 population derived from ‘Yuluxiang’ × ‘Xianghongli’ in the years 2023 and 2024.

Trait	Abbr	Year	Sample Size	Minimum	Maximum	Mean	Standard Deviation	CV/%	Skewness	Kurtosis
Fruit weight/g	FW	2023	140	71.93	364.33	185.10	59.94	32.38	0.48	0.34
		2024	141	57.80	317.53	174.38	49.85	28.59	0.45	0.29
Fruit longitudinal diameter/mm	FLoD	2023	140	49.58	85.67	65.69	8.17	12.44	0.55	0.16
		2024	141	48.63	83.76	64.18	7.95	12.39	0.39	0.49
Fruit lateral diameter/mm	FLaD	2023	140	52.27	92.82	69.74	8.42	12.08	0.38	0.46
		2024	141	51.16	88.61	68.10	8.06	11.83	0.35	0.52
Fruit shape index	FSI	2023	140	0.76	1.14	0.94	0.08	7.96	0.27	0.44
		2024	141	0.75	1.13	0.94	0.07	7.20	0.01	0.19
Total soluble solids/%	TSS	2023	134	9.70	15.50	12.17	1.06	8.69	0.15	0.37
		2024	138	8.94	14.80	11.72	1.11	9.51	0	0.32
Titratable acid/%	TA	2023	136	0.03	0.55	0.19	0.10	54.42	0.32	−0.38
		2024	138	0.05	0.49	0.25	0.10	39.17	0.24	−0.60
Soluble sugar/%	SS	2023	135	3.65	12.95	7.75	1.80	23.25	0.10	−0.09
		2024	137	4.03	12.76	7.91	1.75	22.13	0.06	−0.25
Fruit heart size/mm	FHS	2023	135	17.30	33.61	25.09	3.32	13.22	0.37	−0.19
		2024	136	16.87	33.51	24.57	3.27	13.29	0.43	−0.14
Fruit stalk length/mm	FSL	2023	138	13.60	58.17	35.97	8.45	23.50	0.05	0.01
Leaf longitudinal diameter/mm	LLoD	2023	141	62.84	114.31	88.81	9.02	10.17	−0.07	−0.06
		2024	148	53.93	103.76	73.16	9.73	13.30	0.78	0.94
Leaf lateral diameter/mm	LLaD	2023	141	45.29	79.29	60.88	6.51	10.95	0.08	−0.66
		2024	148	34.57	74.08	50.71	7.39	14.58	0.60	0.39

**Table 3:** Heterosis of fruit-related traits of the F1 population derived from ‘Yuluxiang’ × ‘Xianghongli’.

Trait	Year	Female	Male	MP	Mean	*H*_b_^2^/%	Ta/%	Hm/%	OHP/%	BLP/%
FW/g	2023	256.96	165.47	211.21	185.10	60.47	87.64	−12.36	9.29	54.29
	2024	243.52	136.23	189.88	174.38	55.23	91.84	−8.16	11.35	22.70
FLoD/mm	2023	71.63	56.92	64.28	65.76	27.17	97.84	2.21	20.00	3.57
	2024	70.21	54.36	62.29	64.18	32.31	103.04	3.04	23.40	7.80
FLaD/mm	2023	75.07	75.07	75.07	69.81	61.42	99.52	0.48	27.14	27.14
	2024	72.35	60.68	66.52	68.10	46.78	102.38	−31.90	36.17	21.28
FSI	2023	0.95	0.89	0.92	0.94	34.25	102.23	2.23	41.43	19.29
	2024	0.97	0.90	0.93	0.94	33.21	100.74	0.74	36.17	27.66
TSS/%	2023	12.43	11.20	11.82	12.17	48.83	97.09	2.99	38.81	14.93
	2024	13.02	11.47	12.25	11.72	52.91	95.71	−4.29	12.32	39.13
TA/%	2023	0.20	0.11	0.15	0.19	66.67	80.87	23.65	36.76	20.59
	2024	0.20	0.11	0.16	0.25	51.84	159.24	59.24	60.87	10.14
SS/%	2023	8.68	9.29	8.99	7.75	57.38	116.01	−13.80	18.52	70.37
	2024	8.24	9.17	8.17	7.91	49.25	90.87	−9.13	23.36	58.39
FHS/mm	2023	24.76	26.11	25.43	25.09	56.27	101.37	−1.35	33.33	54.81
	2024	23.39	25.71	24.55	24.57	43.82	100.08	0.08	33.09	42.65
FSL/mm	2023	37.95	18.66	28.31	35.97	85.42	78.69	27.08	40.58	2.17
LLoD/mm	2023	104.32	75.20	89.76	88.81	69.22	101.07	−1.06	4.05	6.76
	2024	101.58	74.31	87.95	73.16	62.81	65.05	−16.81	1.35	58.78
LLaD/mm	2023	72.16	50.15	61.16	60.88	61.27	100.45	−0.45	6.76	4.73
	2024	70.31	51.64	60.98	50.71	55.47	83.17	−16.83	1.35	55.41

**Table 4:** Principal component analysis eigenvalues and contribution of the phenotypic traits.

Principal Component	Eigenvalue	Contribution/%	Cumulative Percentage/%
PCA1	6.346	24.41%	24.41%
PCA2	4.076	15.68%	40.08%
PCA3	2.674	10.28%	50.37%
PCA4	2.226	8.56%	58.93%
PCA5	2.039	7.84%	66.77%
PCA6	1.721	6.62%	73.39%

**Table 5:** Selected eigenvalues and eigenvectors.

Indexes	PCA1	PCA2	PCA3	PCA4	PCA5	PCA6
2023FW	−0.946	0.019	−0.121	0.096	−0.129	0.081
2024FW	−0.945	0.076	−0.100	0.085	−0.107	0.076
2023FLoD	0.059	0.525	0.538	−0.495	0.071	−0.214
2024FLoD	0.062	0.448	0.568	−0.494	0.089	−0.208
2023FLaD	−0.406	−0.718	0.108	−0.019	−0.013	−0.212
2024FLaD	−0.064	−0.244	−0.318	0.166	0.035	−0.191
2023FSI	−0.451	0.307	−0.107	0.373	0.421	0.308
2024FSI	−0.460	0.309	−0.089	0.383	0.410	0.309
PC	0.242	0.120	0.203	0.552	−0.492	−0.146
FT	0.239	0.055	0.175	0.598	−0.473	−0.278
2023FHS	0.042	0.342	0.124	−0.301	−0.067	−0.080
2924FHS	−0.371	−0.730	0.229	−0.031	0.021	−0.273
2023TSS	−0.363	−0.622	0.111	0.002	0.070	−0.433
2024TSS	−0.384	−0.713	0.142	−0.033	0.128	−0.343
2023FSL	−0.247	0.194	−0.446	−0.120	−0.427	−0.210
EFC	−0.148	0.297	−0.427	−0.065	−0.231	−0.261
YLC	−0.016	0.414	−0.469	−0.075	−0.311	−0.159
ABC	0.080	0.403	−0.414	−0.141	−0.315	−0.298
2023LLoD	0.042	0.364	−0.303	0.171	0.592	−0.537
2024LLoD	0.040	0.372	−0.294	0.178	0.589	−0.541
2023LLaD	−0.048	0.387	0.585	0.471	−0.041	−0.150
2024LLaD	−0.018	0.374	0.584	0.478	−0.043	−0.168
2023TA	−0.946	0.019	−0.120	0.096	−0.129	0.080
2024TA	−0.945	0.076	−0.100	0.084	−0.106	0.076
2023SS	0.059	0.524	0.538	−0.495	0.071	−0.214
2024SS	0.062	0.448	0.568	−0.494	0.089	−0.209

## Data Availability

The data can be provided upon reasonable request to the corresponding author.
